# Supplementary material for: Increased risk of near term global warming due to a recent AMOC weakening
Source: Nat Commun. 2021 Oct 20;12:6108. doi: 10.1038/s41467-021-26370-0 (PMC8528826; doi:10.1038/s41467-021-26370-0)
Supplement: Supplementary file 1 — Supplementary Information [file 41467_2021_26370_MOESM1_ESM.pdf]

SUPPLEMENTARY INFORMATION

Increased risk of near term global warming level due to a recent AMOC weakening

Rémy Bonnet, Didier Swingedouw, Guillaume Gastineau, Olivier Boucher, Julie Deshayes, Frédéric Hourdin, Juliette Mignot, Jérôme Servonnat, Adriana Sima

This Supplementary Information provides additional analysis on the attribution of the AMOC weakening to internal variability (Supplementary Note 1) and the relationship between the GSAT and the AMOC over the historical period using three additional ensembles of historical simulations (Supplementary Note 2). Supplementary Figures 1 to 9 and Supplementary Table 1 are also displayed.

Supplementary Note 1. Estimation of the relative influence of internal variability and forced response in the AMOC weakening

In order to investigate and try to attribute the relative influence of internal variability and forced response in the AMOC weakening, we regress the AMOC onto its forced and unforced components (see Methods). This indicates to which extent one or the other component of the AMOC covaries as a function of the total AMOC variations (Supplementary Fig. 1). For all the members, the maximum regression for the forced response is 0.45 Sv Sv^{-1}, with a lot of members having small regression coefficients (mean = 0.15 Sv Sv^{-1}). Therefore, internal climate variability appears to be the main driver of the AMOC variability in the IPSL-EHS over the 1940-2016 period although there appears to be a small forced component in the ensemble mean towards the end of the historical period.

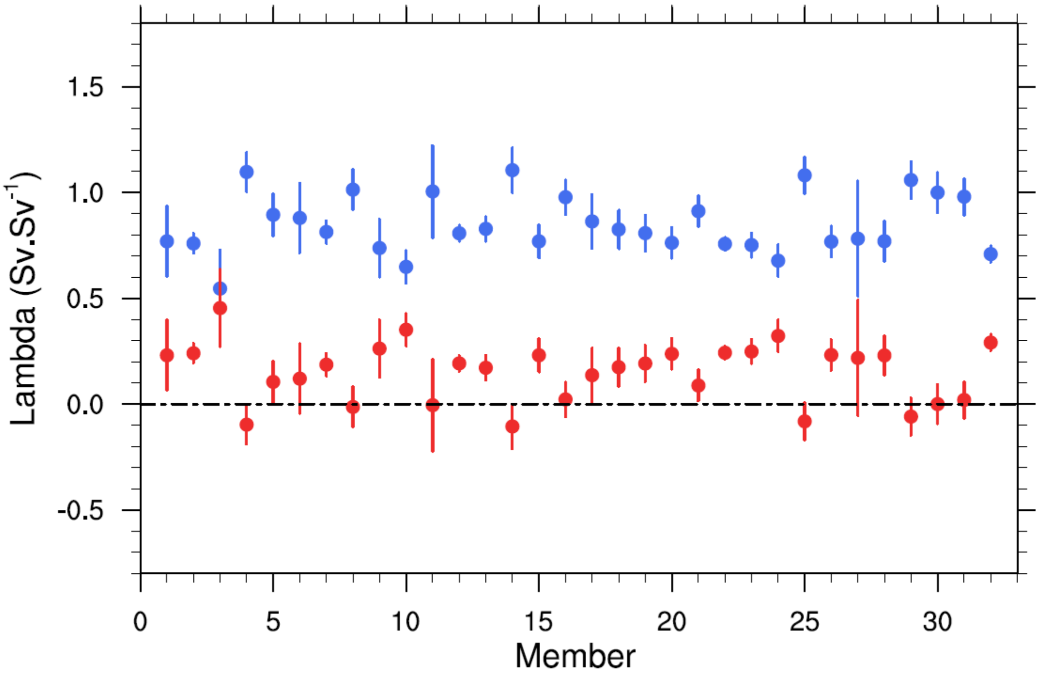

Supplementary Figure 1 | Forced and unforced components to the AMOC. Linear regression coefficient (Sv Sv^{-1}) between the time evolution of the low-pass filtered AMOC strength anomaly in each member of the IPSL_EHS and the forced response of the low-pass

filtered AMOC strength anomaly (red), as well as with the internal variability of the low-pass filtered AMOC strength anomaly (blue). The forced response is defined as the ensemble mean and the internal variability as the difference between the full AMOC time series in each member and the forced response. All time series are considered over the 1940-2016 period. The error bars represent the 95% confidence intervals of the regression coefficient.

Supplementary Note 2. Test of the relationship between the GSAT and the AMOC trends over the recent decades in three other large ensembles

In order to test the relationship found in the IPSL ensemble, we evaluated three other ensembles performed with models with different climate sensitivities (Supplementary Figure 2). The number of models analysed is limited as we can only consider models with publication on the ESGF of at least 30 historical members and the relevant variables needed to estimate the AMOC. The MPI Grand Ensemble[1] (MPI-GE) was performed using the MPI-ESM1.1 model, characterized by a lower low-frequency internal climate variability and a lower effective ECS (2.8 K) than IPSL-CM6A-LR. The two other ensembles available were performed with the CNRM-CM6-1[2] and the CanESM5[3] models, both characterized by relatively high effective ECS values of 4.9 K and 5,6 K, respectively, estimated from an abrupt4xCO_2 experiment.

A positive relationship between the AMOC and GSAT trends is found in the MPI-GE, with the closest members to the observed 1940-2014 GSAT trend exhibiting a negative AMOC trend (Supplementary Fig. 2a). In that respect the MPI-GE is consistent with the IPSL-CM6A-LR model ensemble. The fact that a large fraction of the MPI-GE members have larger GSAT trends than observed despite a low climate sensitivity (effective ECS of 2.8 K) is surprising at first sight. However it should be noted that MPI-ESM1.1 has a fairly weak aerosol forcing of about -0.5 Wm^{-2} according to Mauritsen et al. (2019)[4]. This is in contrast with best estimate of -0.9 Wm^{-2} in chapter 7 of the IPCC Fifth Assessment Report[5] and ranges of -1.6 to -0.6 Wm^{-2} (68% likelihood) and -2.0 to -0.4 Wm^{-2} (90% likelihood) in the authoritative review by Bellouin et al. (2019)[6]. Since then there have been several studies providing observational evidence for a more negative aerosol effective radiative forcing[7–10]. The MPI-ESM1.1 aerosol forcing is also outside the range of aerosol ERF for the subset of CMIP6 models reported in Zanis et al. (2020)[11]. We can thus conclude that matching both the GSAT and AMOC trends in the MPI-GE is only possible because the aerosol forcing is on the low side. In contrast we estimated the aerosol IRF and ERF of IPSL-CM6A-LR to be -0.7 and -0.6 Wm^{-2}, which is not very strong for a large ECS model. Had the MP-ESM1.1 a stronger (more negative) aerosol ERF, it would not have been possible for the MPI-GE to match both the observed GSAT and AMOC trends. The analysis of the MPI-GE therefore does not invalidate our hypothesis.

The CNRM ensemble is characterized by a very strong positive relationship between the AMOC and the GSAT trends since 1940 and a larger low-frequency natural variability than the other ensembles (Supplementary Fig. 2b). Some historical members match both the GSAT and the AMOC 1940-2014 trends, and the members with the lowest RMSE over the 1900-2014 are among these members. The CNRM-CM6-1 model could have been compatible with an even lower observed GSAT trend despite its rather large ECS of 4.9 K. This model thus supports our hypothesis with IPSL-CM6A-LR.

Finally, for the CanESM5 ensemble, the GSAT and AMOC trends over the 1940-2014 period show a weak relationship (Supplementary Fig. 2c). None of the historical simulations is close to the observations in terms of the GSAT trend. Given that this model has little natural variability, it does not invalidate our hypothesis though not much can be concluded.

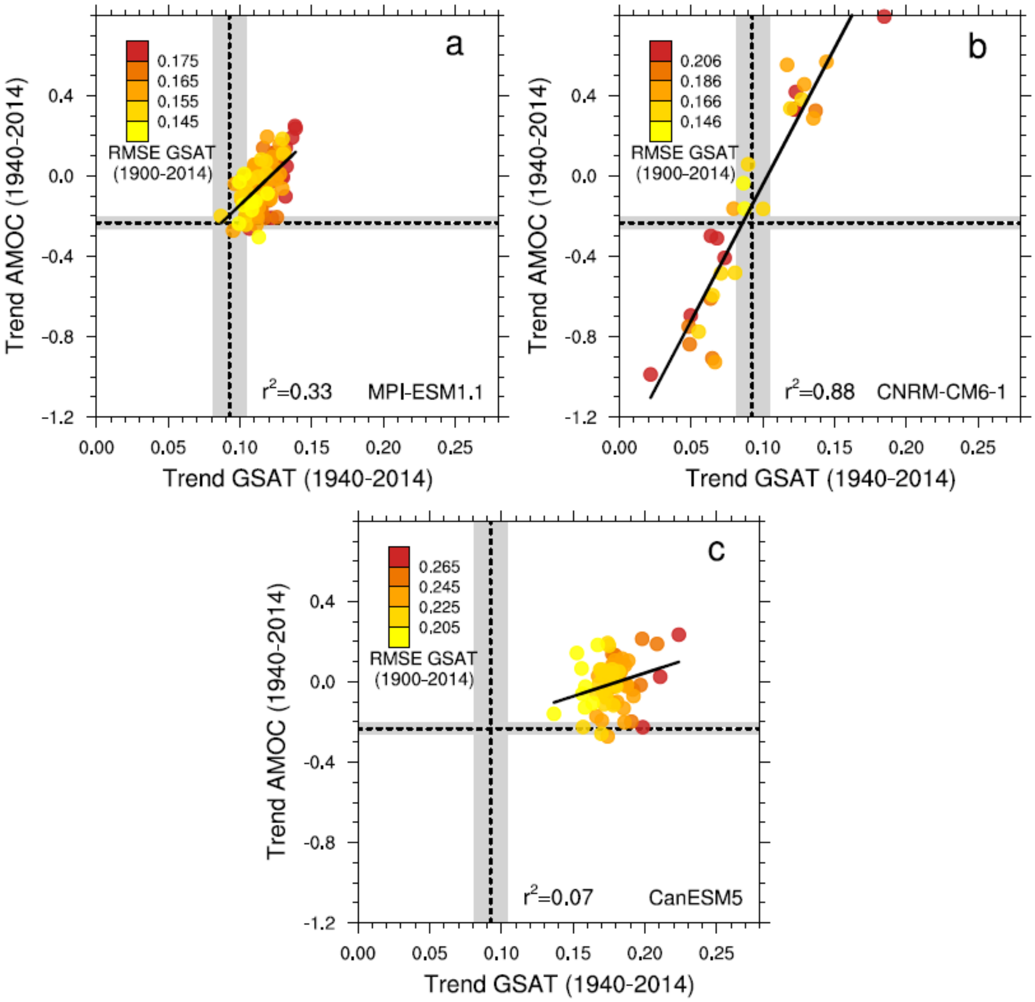

Supplementary Figure 2 | Relationship between GSAT and AMOC over the historical period. Scatter plot of GSAT (K decade^{-1}) versus AMOC at 26°N (Sv decade^{-1}) trends calculated over the 1940-2014 period from **(a)** the MPI ensemble (100 members), **(b)** the CNRM ensemble (30 members) and **(c)** the CanESM5 ensemble (65 members) of historical simulations. We show all the ensemble members (filled circles) and the observations (horizontal and vertical dashed lines). The observations shown are HadCRUT4-CW[12,13] for the GSAT and the Caesar index[14] as a proxy for the AMOC evolution, with the related uncertainties (gray). The AMOC time series from the MPI-GE are the AMOC at 26°N and 1000 m depth provided in Marotzke (2019)[15].

Additional Supplementary Figures 3 to 9

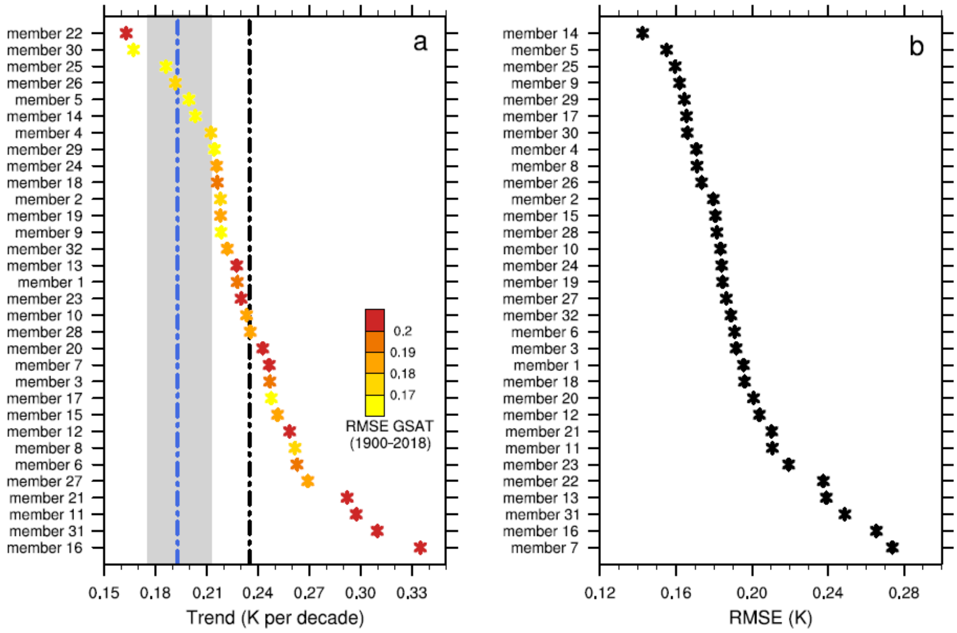

Supplementary Figure 3 | Trends and RMSE of the GSAT across the IPSL-EHS. (a), Trends in the Global near-Surface Air Temperature (GSAT, K decade^{-1}) calculated over the 1970-2018 period for the HadCRUT4-CW[12,13] dataset (blue dot-dashed line), the Berkeley[16] dataset (brown dot-dash line, overlying the blue dot-dashed line), the ensemble mean of the historical simulations (black dot-dashed line) and the 32 members (stars) ranked from the weakest to strongest trend. The colors of the stars display the RMSE of the GSAT (K) over the 1900-2018 period as per the color scale on the right, with respect to the HadCRUT4-CW dataset. (**b),** Root mean square error (RMSE) of the GSAT anomaly of the individual members of the IPSL-EHS against the HadCRUT4-CW dataset over the 1900-2018 period.

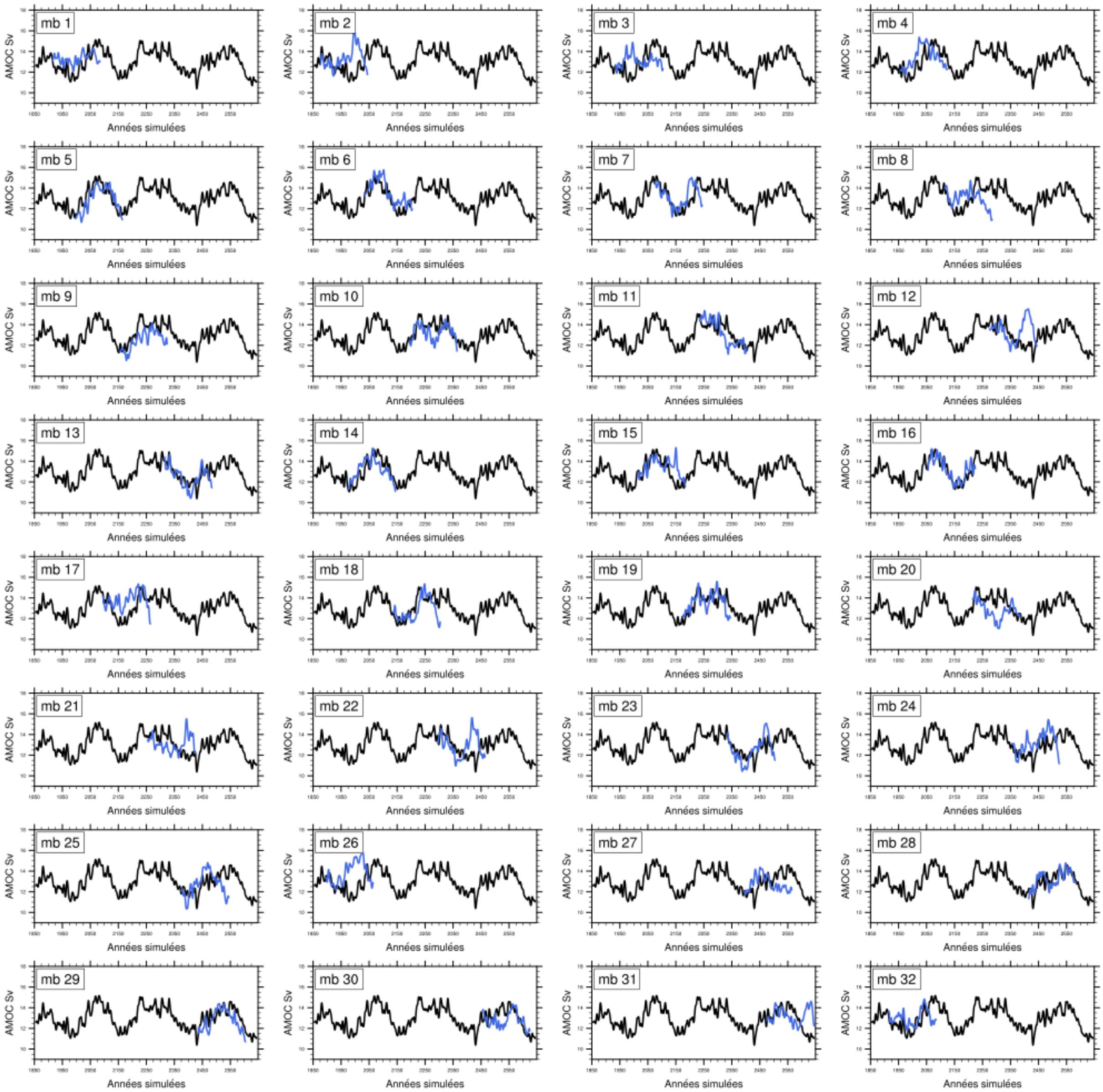

Supplementary Figure 4 | Comparison of the AMOC time evolution in the IPSL-EHS relative to the initial date in the pre-industrial control simulation. Time evolution of the low-pass filtered AMOC (Sv) from the *piControl* simulation (black) and from the 32 historical simulations (blue) from the IPSL-EHS ranked from the first to the last member. A lanczos low-pass filter with a cutoff period of 11 years is used.

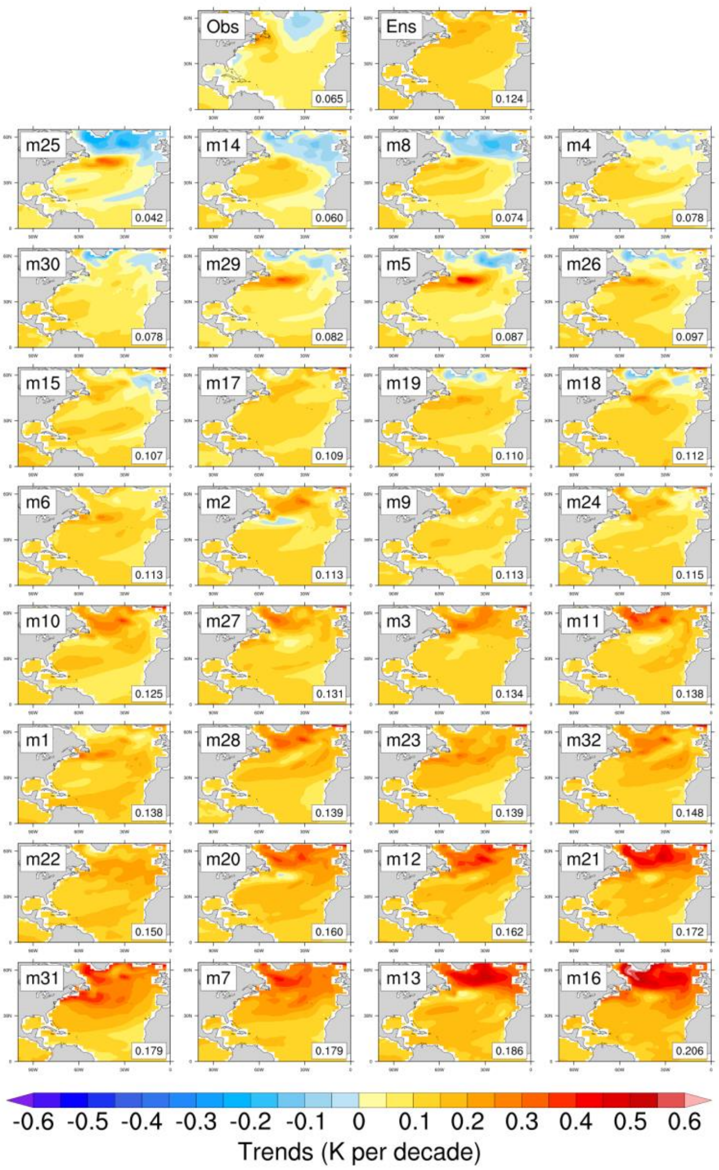

Supplementary Figure 5 | SST trends over the North Atlantic in the IPSL-EHS. Trends in the North Atlantic Ocean near surface air temperature (K decade^{-1}) over the 1940–2018 period from the ERSSTv5[17] observed dataset (top left panel), from the IPSL-EHS, with the ensemble mean (top right) and the 32 members ranked from the lowest (top left) to the highest trend (bottom right). The member number and the basinwide trend values, in K decade^{-1}, are indicated on the top-left and bottom-right corners of each panel, respectively.

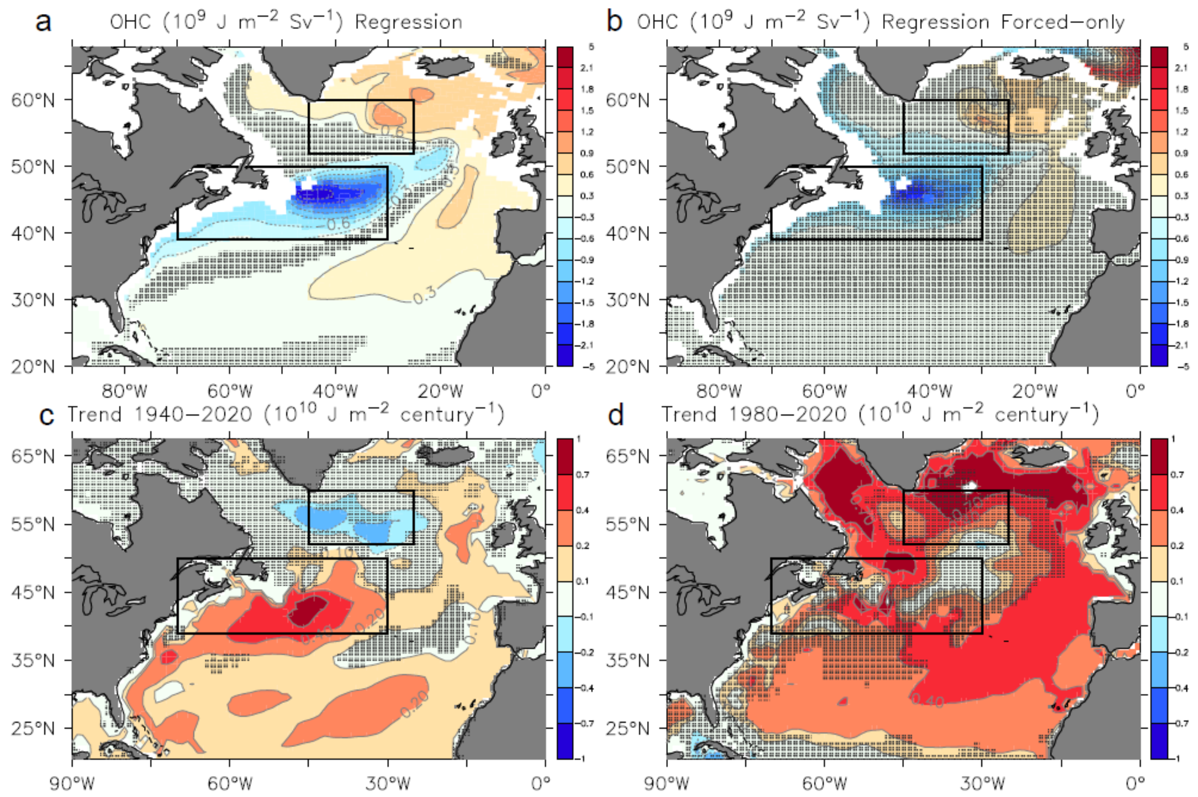

Supplementary Figure 6 | Estimation of the AMOC fingerprint on upper OHC. (a), Regression of the upper Ocean Heat Content (0-700 m) onto the AMOC from the 32 members of the IPSL-EHS put end to end. **(b),** Same as **a** but using the forced signal of the AMOC (ensemble mean). **(c),** Trend in the upper Ocean Heat Content (0-700 m) calculated over the 1940-2020 period from the IAP observed dataset[18,19]. **(d),** Same as **c** but for the trend calculated over the 1980-2020 period. The areas where the trends or regressions are significant taking serial autocorrelation into account[20] (p-value<0.05) are the unshaded areas. The rectangles indicate the areas used for the calculation of the AMOC fingerprint based on the upper Ocean Heat Content (0-700 m).

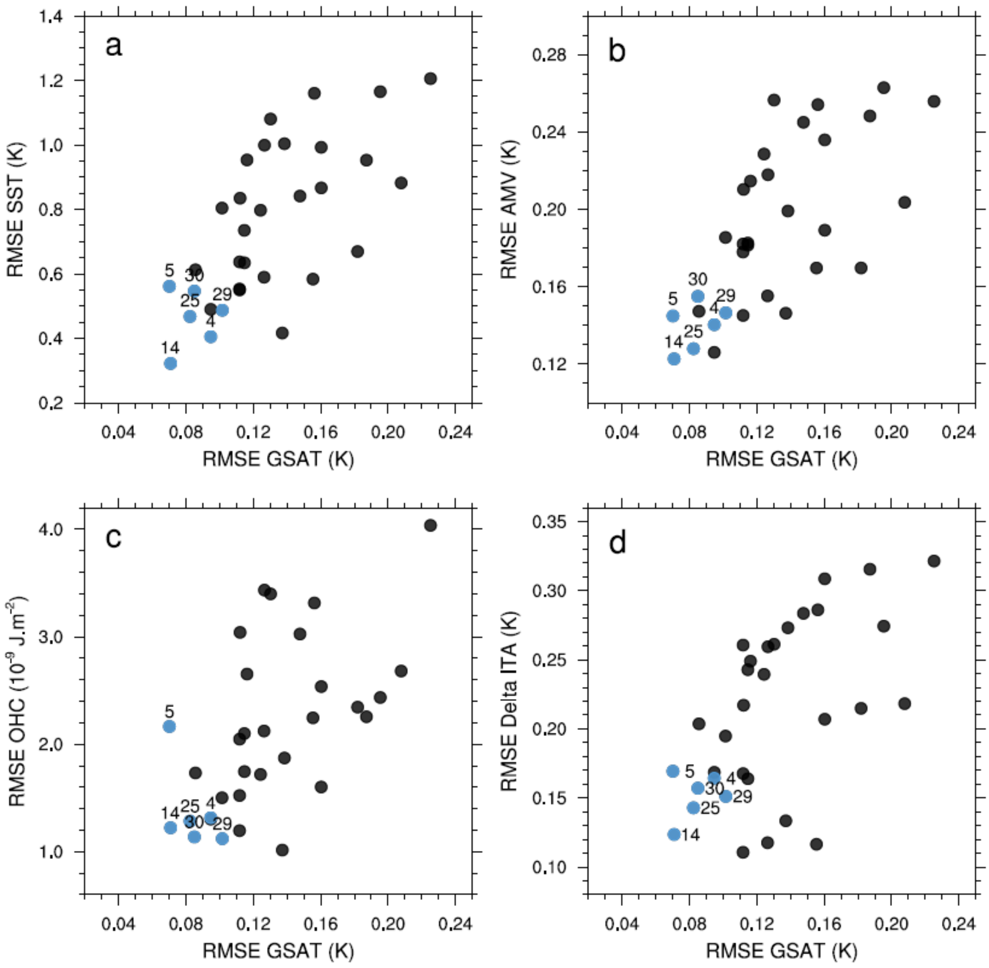

Supplementary Figure 7 | IPSL-CM6A-LR model error as compared to four different observed AMOC fingerprints against the model error to the observed GSAT anomaly over the historical period. (a), Scatterplot between the root mean square error (RMSE) of the Caesar SST index[14] (K) against the RMSE of the GSAT anomaly calculated over the 1900-2018 period for each member of the IPSL-EHS, with the subset of members (14, 5, 25, 29, 4, 30) highlighted Fig 2a in blue. Members in the lower left-hand corner are the most consistent with the observations and conversely. **(b)**, Same as panel **(a)** but for the AMV index. **(c),** Same as panel **(a)** but for an index based on the Ocean Heat Content (OHC) anomaly (10^9 J.m^{-2}) between 0 and 700 m averaged over the Newfoundland region minus the OHC averaged over the North Atlantic Subpolar Gyre region. **(d),** Same as panel **(a)** but for the ΔITA (K) index. The RMSE of the GSAT is computed against the HadCRUT4-CW[12,13] dataset. For the Caesar index, the RMSE of the North Atlantic Subpolar Gyre SST is computed against the ERSSTv5 dataset[17]. The OHC index is based on the IAP observational dataset[18,19]. See the main text for a full description of the AMOC indices.

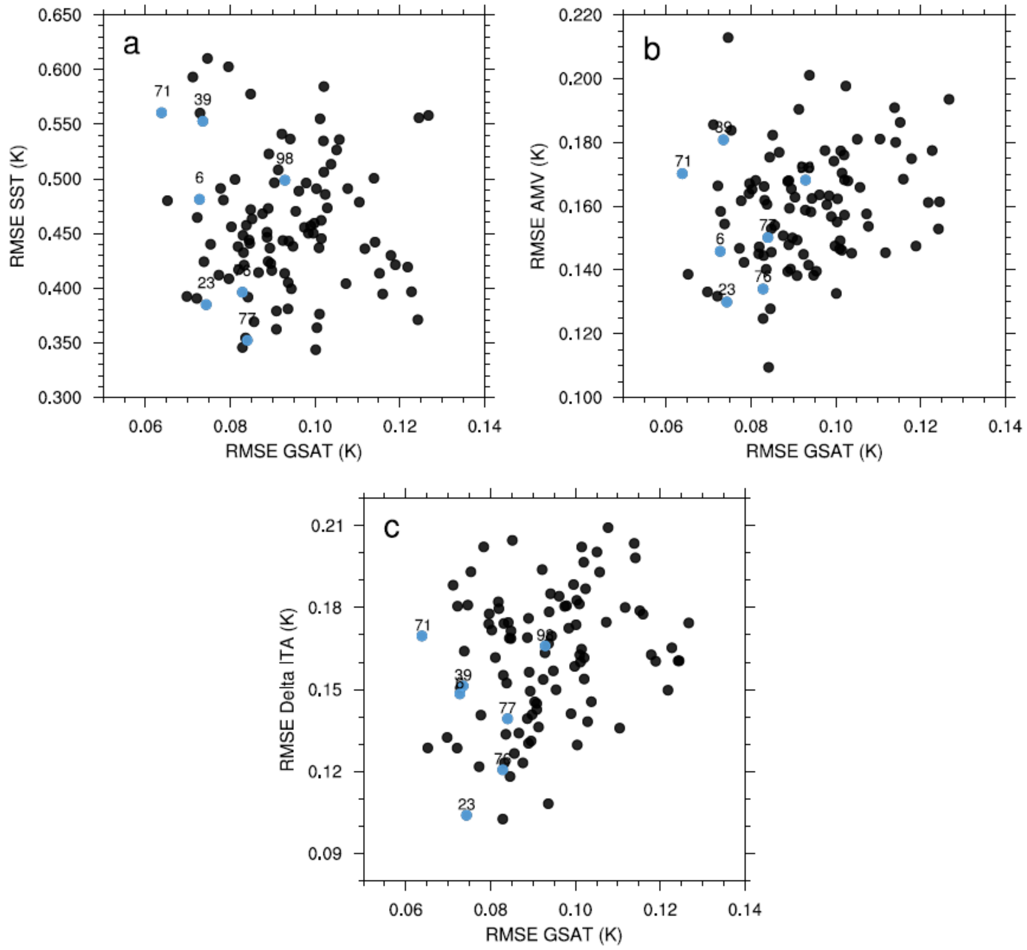

Supplementary Figure 8 | Same as Figure S7 but for the MPI ensemble. (a) Scatterplot between the root mean square error (RMSE) of the Caesar SST index[14] (K) against the RMSE of the GSAT anomaly for each member of the MPI-GE calculated over the 1900-2014 period. **(b)**, Same as panel **(a)** but for the AMV index. **(c),** Same as panel **(a)** but for the ΔITA (K) index. See the main text for a full description of the AMOC indices. The subset of members that are labelled in blue (6, 23, 39, 71, 76, 77, 98) corresponds to the members with GSAT trends lower than 0.11 K per decade and with AMOC weakening between -0.115 and -0.4 Sv per decade, as well as a RMSE of GSAT lower than 0.155 (Supplementary Fig. 2). Members in the lower left-hand corner are the most consistent with the observations and conversely.

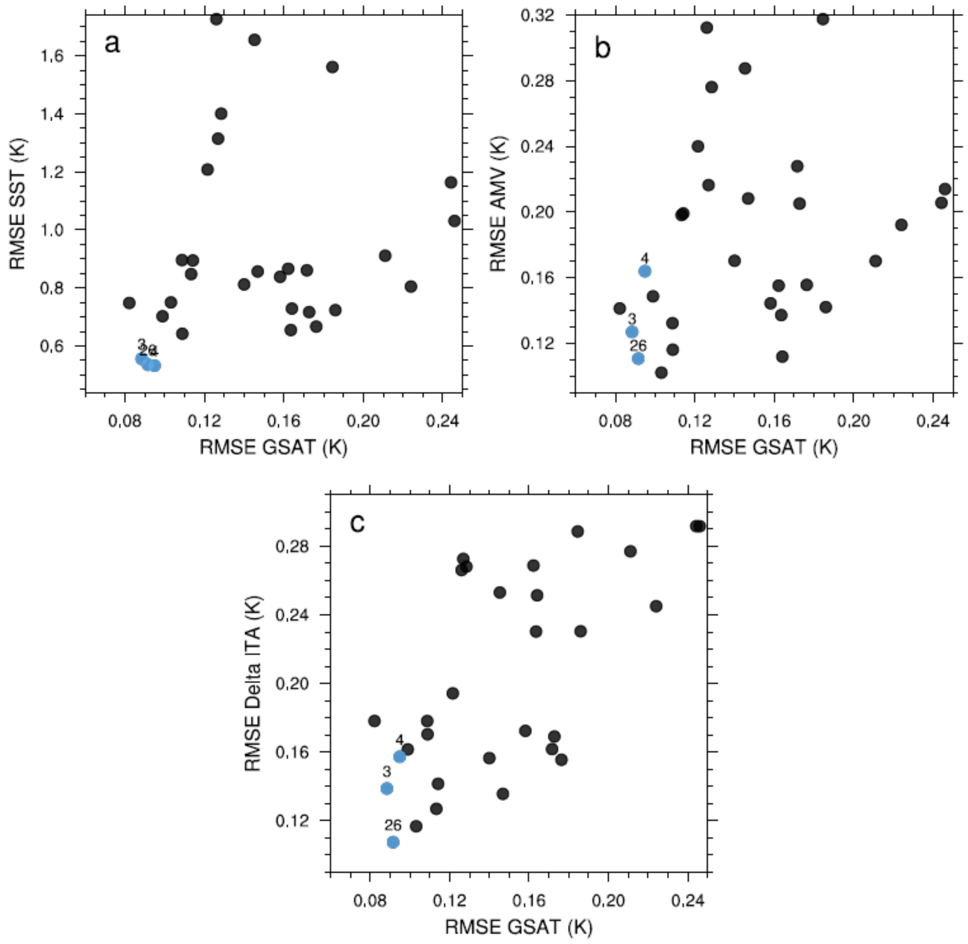

Supplementary Figure 9 | Same as Figure S7 but for the CNRM ensemble. The subset of members that are labelled in blue (3, 4 and 26) corresponds to the three members with the closest GSAT and AMOC trends to the observations, as well as the lowest RMSE (Fig. Supplementary 2). Members in the lower left-hand corner are the most consistent with the observations and conversely.

Supplementary Table 1: Simulations and models used.

Model name	Nominal Resolution		Duration of *piControl* used (years)	Number of *historical* used	ECS[21] (in K)	Forcings/ Reference
	Atmosphere	Ocean				
IPSL-CM6A-LR	250 km L79	100 km L75	500	32	4.56	CMIP6[22]
CNRM-CM6-1	250 km L91	100 km L75	500	30	4.90	CMIP6[2]
CNRM-ESM2-1	250 km L91	100 km L75	500	-	4.79	CMIP6[23]
MPI-ESM1-1 modified for the MPI-GE	250 km L47	250 km L40	-	100	2.8	CMIP5[1]
MPI-ESM1-2-HR	100 km L95	50 km L40	500	-	2.98	CMIP6[24]
CESM2-FV2	250 km L30	100 km L60	500	-	-	CMIP6[25]
CESM2-WACCM	100 km L70	100 km L60	500	-	4.68	CMIP6[25]
SAM0-UNICON	100 km L30	100 km L60	500	-	3.72	CMIP6[26]
CMCC-CM2	100 km L30	100 km L50	500	-	-	CMIP6[27]
MRI-ESM2	100 km L80	100 km L61	500	-	3.13	CMIP6[28]
EC-Earth3	100 km L91	100 km L75	500	-	4.10	CMIP6[29]
CanESM5	500 km L49	100 km L45	500	65	5.64	CMIP6[3]
NorCPM1	250 km L26	100 km L53	500	-	-	CMIP6[30]
CIESM	100 km L30	50 km L46	500	-	5.67	CMIP6[31]

Supplementary References

1. Maher, N. *et al.* The Max Planck Institute Grand Ensemble: enabling the exploration of climate system variability. *J. Adv. Model. Earth Syst.* **11**, 2050–2069 (2019).
2. Voldoire, A. *et al.* Evaluation of CMIP6 DECK experiments with CNRM-CM6-1. *J. Adv. Model. Earth Syst.* **11**, 2177–2213 (2019).
3. Swart, N. C. *et al.* The Canadian Earth System Model version 5 (CanESM5.0.3). *Geosci. Model Dev.* **12**, 4823–4873 (2019).
4. Mauritsen, T. *et al.* Developments in the MPI-M Earth System Model version 1.2 (MPI-ESM1.2) and Its Response to Increasing CO2. *J. Adv. Model. Earth Syst.* **11**, 998–1038 (2019).
5. Boucher, O. et al. *Clouds and Aerosols*. *Climate change 2013 : the physical science basis : Working Group I contribution to the fifth assessment report of the Intergovernmental Panel on Climate Change* 571–657 https://www.research-collection.ethz.ch/handle/20.500.11850/78882 (2013).
6. Bellouin, N. *et al.* Bounding global aerosol radiative forcing of climate change. *Rev. Geophys.* **58**, e2019RG000660 (2020).
7. Hasekamp, O. P., Gryspeerdt, E. & Quaas, J. Analysis of polarimetric satellite measurements suggests stronger cooling due to aerosol-cloud interactions. *Nat. Commun.* **10**, 5405 (2019).
8. McCoy, I. L. *et al.* The hemispheric contrast in cloud microphysical properties constrains aerosol forcing. *Proc. Natl. Acad. Sci.* **117**, 18998–19006 (2020).
9. Diamond, M. S., Director, H. M., Eastman, R., Possner, A. & Wood, R. Substantial cloud brightening from shipping in subtropical low clouds. *AGU Adv.* **1**, e2019AV000111 (2020).
10. Christensen, M. W., Jones, W. K. & Stier, P. Aerosols enhance cloud lifetime and brightness along the stratus-to-cumulus transition. *Proc. Natl. Acad. Sci.* **117**, 17591–17598 (2020).

11. Zanis, P. *et al.* Fast responses on pre-industrial climate from present-day aerosols in a CMIP6 multi-model study. *Atmospheric Chem. Phys.* **20**, 8381–8404 (2020).
12. Jones, P. D. *et al.* Hemispheric and large-scale land-surface air temperature variations: An extensive revision and an update to 2010. *J. Geophys. Res. Atmospheres* **117**, (2012).
13. Cowtan, K. & Way, R. G. Coverage bias in the HadCRUT4 temperature series and its impact on recent temperature trends. *Q. J. R. Meteorol. Soc.* **140**, 1935–1944 (2014).
14. Caesar, L., Rahmstorf, S., Robinson, A., Feulner, G. & Saba, V. Observed fingerprint of a weakening Atlantic Ocean overturning circulation. *Nature* **556**, 191–196 (2018).
15. Marotzke, J. Quantifying the irreducible uncertainty in near-term climate projections. *WIREs Clim. Change* **10**, e563 (2019).
16. Rohde, R., Muller, R., Jacobsen, R., Perlmutter, S. & Mosher, S. Berkeley Earth temperature averaging process. *Geoinformatics Geostat. Overv.* **01**, (2013).
17. Huang, B. *et al.* Extended Reconstructed Sea Surface Temperature, Version 5 (ERSSTv5): upgrades, validations, and intercomparisons. *J. Clim.* **30**, 8179–8205 (2017).
18. Cheng, L. & Zhu, J. Benefits of CMIP5 multimodel ensemble in reconstructing historical ocean subsurface temperature Variations. *J. Clim.* **29**, 5393–5416 (2016).
19. Cheng, L. *et al.* Improved estimates of ocean heat content from 1960 to 2015. *Sci. Adv.* **3**, e1601545 (2017).
20. Ebisuzaki, W. A method to estimate the statistical significance of a correlation when the data are serially correlated. *J. Clim.* **10**, 2147–2153 (1997).
21. Zelinka, M. D. *et al.* Causes of higher climate sensitivity in CMIP6 models. *Geophys. Res. Lett.* **47**, e2019GL085782 (2020).
22. Boucher, O. *et al.* Presentation and evaluation of the IPSL-CM6A-LR climate model. *J. Adv. Model. Earth Syst.* **12**, e2019MS002010 (2020).
23. Séférian, R. *et al.* Evaluation of CNRM Earth System Model, CNRM-ESM2-1: role of Earth system processes in present-day and future climate. *J. Adv. Model. Earth Syst.* **11**,

4182–4227 (2019).

24. A. Muller, R., Rohde, R., Jacobsen, R., Muller, E. & Wickham, C. A new estimate of the average Earth surface land temperature spanning 1753 to 2011. *Geoinformatics Geostat. Overv.* **01**, (2013).

25. Danabasoglu, G. *et al.* The Community Earth System Model Version 2 (CESM2). *J. Adv. Model. Earth Syst.* **12**, e2019MS001916 (2020).

26. Park, S., Shin, J., Kim, S., Oh, E. & Kim, Y. Global Climate Simulated by the Seoul National University Atmosphere Model Version 0 with a Unified Convection Scheme (SAM0-UNICON). *J. Clim.* **32**, 2917–2949 (2019).

27. Cherchi, A. *et al.* Global mean climate and main patterns of variability in the CMCC-CM2 coupled model. *J. Adv. Model. Earth Syst.* **11**, 185–209 (2019).

28. Seiji, Y. *et al.* The Meteorological Research Institute Earth System Model version 2.0, MRI-ESM2.0: description and basic evaluation of the physical component. *気象集誌 第2輯* **advpub**, (2019).

29. Doescher, R. The EC-Earth Consortium: The EC-Earth3 Earth System Model for the Climate Model Intercomparison Project 6.". *Manuscr. Prep.* (2020).

30. Bethke, I. *et al.* NCC NorCPM1 model output prepared for CMIP6 CMIP. (2019) doi:10.22033/ESGF/CMIP6.10843.

31. Lin, Y. *et al.* Community Integrated Earth System Model (CIESM): description and evaluation. *J. Adv. Model. Earth Syst.* **12**, e2019MS002036 (2020).
